# Introducing a new method for classifying skull shape abnormalities related to craniosynostosis

**DOI:** 10.1007/s00431-020-03643-2

**Published:** 2020-04-17

**Authors:** Otto D. M. Kronig, Sophia A. J. Kronig, Henri A. Vrooman, Jifke F. Veenland, Mariëlle Jippes, Terence Boen, Léon N. A. Van Adrichem

**Affiliations:** 1 Department of Plastic and Reconstructive Surgery and Hand Surgery, Dutch Craniofacial Centre, Erasmus MC - Sophia Children’s Hospital, University Medical Centre Rotterdam, Rotterdam, The Netherlands; 2 Department of Plastic and Reconstructive Surgery and Hand Surgery, University Medical Centre Utrecht, Heidelberglaan 100, 3584 CX Utrecht, The Netherlands; 3 Department of Radiology, Erasmus MC, Rotterdam, University Medical Centre Rotterdam, Rotterdam, The Netherlands; 4 Department of Medical Informatics, Erasmus MC, Rotterdam, University Medical Centre Rotterdam, Rotterdam, The Netherlands

**Keywords:** Craniosynostosis, Shape analysis, Reliability, Computer-assisted diagnosis, Computer tomography

## Abstract

We present a novel technique for classification of skull deformities due to most common craniosynostosis. We included 5 children of every group of the common craniosynostoses (scaphocephaly, brachycephaly, trigonocephaly, and right- and left-sided anterior plagiocephaly) and additionally 5 controls. Our outline-based classification method is described, using the software programs OsiriX, MeVisLab, and Matlab. These programs were used to identify chosen landmarks (porion and exocanthion), create a base plane and a plane at 4 cm, segment outlines, and plot resulting graphs. We measured repeatability and reproducibility, and mean curves of groups were analyzed. All raters achieved excellent intraclass correlation scores (0.994–1.000) and interclass correlation scores (0.989–1.000) for identifying the external landmarks. Controls, scaphocephaly, trigonocephaly, and brachycephaly all have the peak of the forehead in the middle of the curve (180*°*). In contrary, in anterior plagiocephaly, the peak is shifted (to the left of graph in right-sided and vice versa). Additionally, controls, scaphocephaly, and trigonocephaly have a high peak of the forehead; scaphocephaly has the lowest troughs; in brachycephaly, the width/frontal peak ratio has the highest value with a low frontal peak.

*Conclusion*: We introduced a preliminary study showing an objective and reproducible methodology using CT scans for the analysis of craniosynostosis and potential application of our method to 3D photogrammetry.

**Table:** 

**What is Known:** *• Diagnosis of craniosynostosis is relatively simple; however, classification of craniosynostosis is difficult and current techniques are not widely applicable.*	
**What is New:** *• We introduce a novel technique for classification of skull deformities due to craniosynostosis, an objective and reproducible methodology using CT scans resulting in characteristic curves. The method is applicable to all 3D-surface rendering techniques.* *• Using external landmarks and curve analysis, specific and characteristic curves for every type of craniosynostosis related to the specific skull deformities are found.*

**What is Known:**

*• Diagnosis of craniosynostosis is relatively simple; however, classification of craniosynostosis is difficult and current techniques are not widely applicable.*

**What is New:**

*• We introduce a novel technique for classification of skull deformities due to craniosynostosis, an objective and reproducible methodology using CT scans resulting in characteristic curves. The method is applicable to all 3D-surface rendering techniques.*

*• Using external landmarks and curve analysis, specific and characteristic curves for every type of craniosynostosis related to the specific skull deformities are found.*

## Introduction

In normal skull development, the cranial sutures allow the brain to expand as the infant matures. In craniosynostosis patients, one or more sutures have prematurely fused to form a solid bone connection, resulting in a restriction of expansion of the cranial vault, normal growth of the brain, and deformation of the calvaria [1]. Craniosynostosis is usually diagnosed upon clinical judgment (medical history and physical examination, including anthropometry) and is confirmed by radiographic imaging.

Classification of skull shape deformities is essential and could allow for type and severity to be classified and thus may aid in clinical as well as research applicability to evaluate presentation, development, and treatment [2–7].

A variety of methods to diagnose and measure skull shape, and additionally craniosynostosis, are available; some of the currently used methods are cephalic index (CI), head circumference, intracranial volume (ICV), scaphocephaly severity index (SSI), cranial vault asymmetry index (CVAI), and the plagiocephalometry (PCM) [3, 6, 8–11]. However, these methods are not capturing the different aspects of skull shape deformity (CI, head circumference, and ICV) and are solely applicable for one specific type of craniosynostosis or positional skull deformities (SSI, CVAI, and PCM).

In this study, we will propose an outline-based method applicable for each type of craniosynostosis. A method based on the outline of the skull has the advantage of capturing the actual skull shape variation. External landmarks (soft tissue landmarks, visible with the bare eye) will be used to extract an outline of the skull shape using CT scans, resulting in sinusoid curves. These curves will be assessed for different variables specific for the most common types of craniosynostosis. Our hypothesis is that by using external landmarks in combination with the outline-based objective analysis, we are able to capture the clearly visible characteristics of craniosynostosis by all methods of 3D imaging (independent of CT imaging), enabling a repeatable and objective analysis of skull shape changes during growth and treatment.

## Material and methods

### Patients

For validation of external landmarks, we included 26 children (age < 1 year). We included 24 patients with nonsyndromic craniosynostosis (11 scaphocephaly; 11 trigonocephaly; 1 brachycephaly; and 1 left-sided plagiocephaly anterior) and 2 control patients.

For analysis of the sinusoid curves, we included 25 children (age < 1 year) with nonsyndromic craniosynostosis. Five children of every type of most common craniosynostosis (scaphocephaly, brachycephaly, trigonocephaly, and right- and left-sided (RS and LS) anterior plagiocephaly) were included. In addition, 5 control patients were included.

For all craniosynostosis patients, a preoperative CT scan of the head needed to be available. Children with other congenital or traumatic craniofacial malformations, including multiple suture craniosynostosis, facial fractures, or soft tissue swelling, were excluded. Used CT scans were part of routine diagnostic evaluation in patients suspected for craniosynostosis.

To be eligible as control patient, the CT scan needed to be made at an age of 6 years or younger and needed to contain orbits and ears. These patients underwent CT scanning for possible neurotrauma. The scans were negative for congenital or traumatic craniofacial malformations, including craniosynostosis, facial fractures, or soft tissue swelling.

The patients were diagnosed at the Erasmus Medical Centre, Sophia Children’s Hospital Rotterdam, a specialized center for treatment of a variety of skull deformities.

The study was approved by the local Medical Ethics Review Committee (MEC-2016-467). The study was deemed a retrospective clinical study and did not require formal research ethics approval under the Medical Research Involving Human Subjects Act (WMO).

### Repeatability and reproducibility of external landmarks

**Fig. 1:**
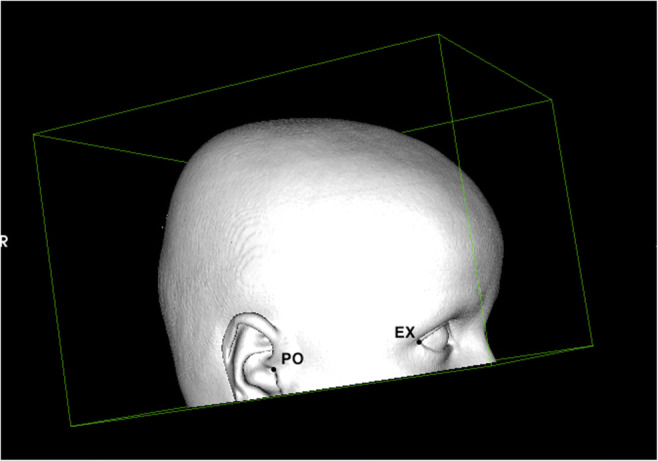
The exocanthion and porion as anatomical landmarks (view from the right)

**Fig. 2:**
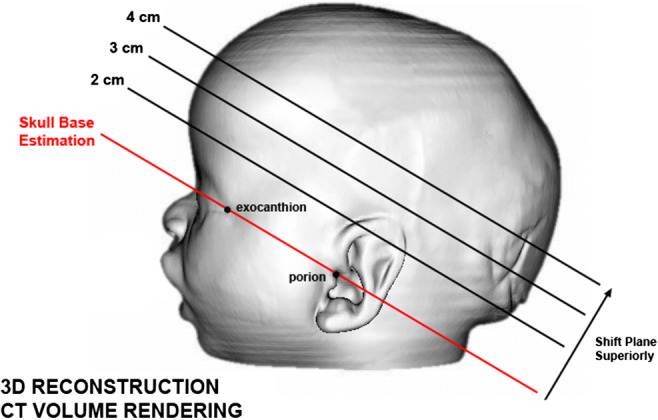
Visualization of the landmarks and different heights of the planes

Four external anatomic landmarks were located and marked by three different individuals, a plastic surgeon and two medical students: left and right exocanthion (ex) and left and right porion (po) (Figs. 1 and 2). This was repeated twice in different settings to get a total of three ratings per landmark, per rater, and per sample. The *x*, *y*, and *z* coordinates of all ratings were recorded. To ensure repeatability and reproducibility of the external landmarks, intra- and interrater reliability were calculated.

### Methodology for creating sinusoid curves

**Fig. 3:**
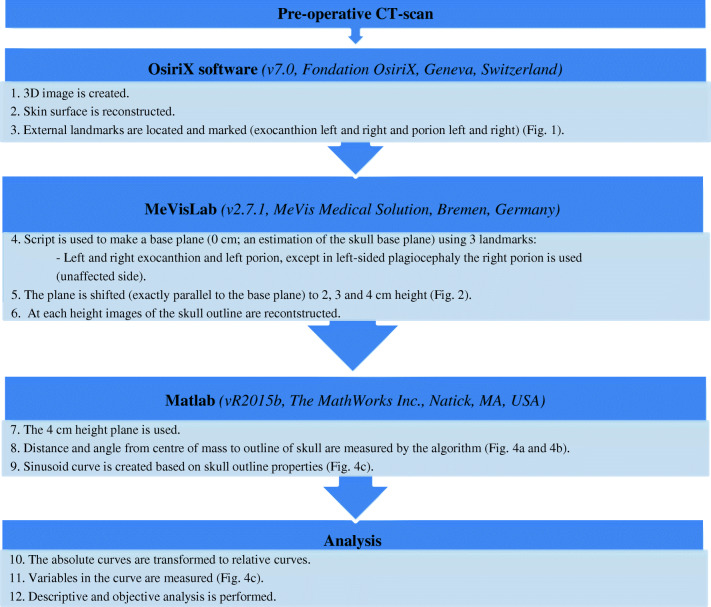
Summary of methods

**Fig. 4:**
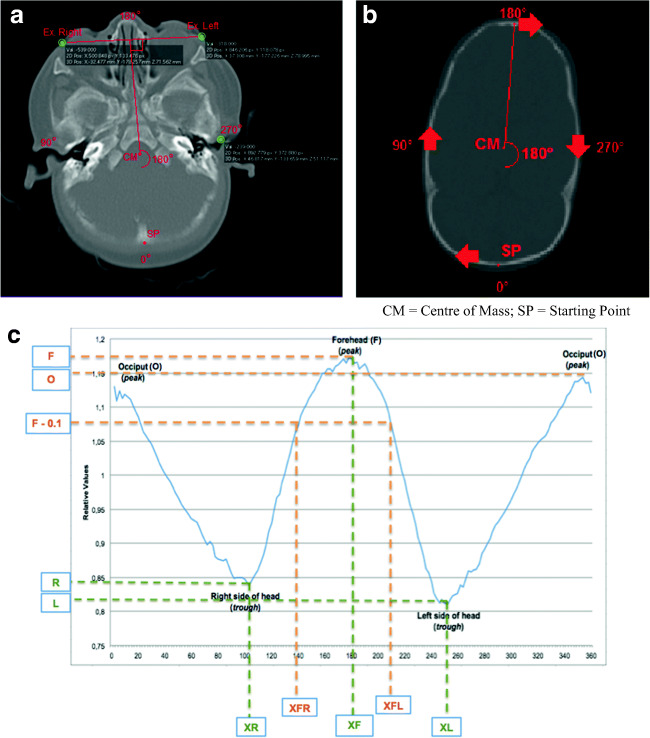
Visualization of the starting point of the curve and the resulting sinusoid curve; the outline was made with the slices shown in Fig. 4b. **a** Plane on 0 cm height; this figure shows how the starting point of the curve is determined. Also, the degrees of the circle/outline are added, which are represented in the curve. **b** Plane on 4 cm height; this figure shows the starting point and the direction of the curve. **c** The resulting curve. The different variables are marked. Curve starts at the occiput (SP; Fig. 4b) and follows the skull outline (on CT scan) clockwise; therefore, the first trough represents the right side of the head. The second peak is the forehead; the second trough is the left side of the head. Curve stops where it started (at the occiput).

Figure 3 shows the steps to create a sinusoid curve using external landmarks on CT scans. Figure 4 shows a visualization of the process. A requirement for defining a base plane in three dimensions is having three landmarks (with *x*, *y*, and *z* coordinate). We identified four landmarks; however, for the purposes of this study, we used the following combination of landmarks: left and right exocanthion and left porion, except in left-sided plagiocephaly, the right porion was used. The plane 4 cm higher than the basal plane was analyzed.

The center of mass (CM) on the 4 cm height planes is computed. A two-dimensional coordinate system is formed in which a distance from a reference point and an angle from a reference direction determine each point on a plane. Two (virtual) lines are drawn: one line connecting the left and right ex (Fig. 4a), and a second (virtual) line perpendicular at the middle of the first line. The point where the second line intersects the occiput determines the starting point of the curve.

Figure 4c shows a graphic presentation of the involved measurements and resulting plot. The sinusoid corresponds to the shape of the skull outline and is the foundation for craniosynostosis analysis.

Absolute curves of the skull outlines were transformed to relative curves in order to achieve results independent of skull size and therefore age. A value of 1.0 represents the mean value, and values lower or higher than 1.0 represent a value lower respectively higher than the mean value. The *x*-axis of the plot shows the angle from a reference direction; the *y*-axis shows relative values of the distance from the CM. This information will result in a sinusoid curve (Fig. 4c).

### Analysis

Table 1 shows the obtained and specific values extracted from the curves; these values are used for analysis of the curves specific for each type of craniosynostosis.

**Table 1:** Extracted and calculated variables from curve

Length and width			
Variable	Abbreviation	Variable	Abbreviation
Maximum value of forehead peak	F	Minimum value of the right side of the head (trough)	R
Maximum value of occiput peak	O	Minimum value of the left side of the head (trough)	L
Calculations			
Calculated variable	Formula	Calculated variable	Formula
Mean of both sides	R/2 + L/2	Total maximum length	F + O
Difference of the occiput and sides	O-(R/2-L/2)	Difference of the forehead and sides	F-(R/2-L/2)
Total minimum value in curve of width (max. clinical width)	R + L	Minimum value (in curve of) width/maximum length ratio (comparable with cranial index)	(R + L)/(F + O)
Difference of the forehead and occiput	F-O	Ratio length to width of the skull	(F + O)/(R + L)
Forehead shape analysis			
Variable	Abbreviation	Variable	Abbreviation
*X*-value (in degrees) of the maximum forehead value	XF	*X*-value (in degrees) for maximum forehead minus 0.1 (F-0.1) on the left side	XFL
*X*-value (in degrees) for maximum forehead minus 0.1 (F-0.1) on the right side	XFR		
Calculations			
Calculated variable	Formula		
Width of the forehead	(XFL-XFR)/(F-0.1)		
(A)symmetry analysis			
Variable	Abbreviation	Variable	Abbreviation
*X*-value (in degrees) of minimum value of width on right side	XR	*X*-value (in degrees) of minimum value of width on the left side	XL
Calculations			
Calculated variable	Formula		
Asymmetry ratio	(XF-XR)/(XL-XF)		

For each type of skull shape, the mean, minimum, and maximum values were established for all curves and for extracted and calculated values. Mean difference of the *y*-value for each patient group for every degree (*x*-axis) is calculated. We determine if the peak of the forehead is at 180° ± 12.

### Statistical analysis

Statistical analyses were performed using the SPSS for Windows (Version 21, SPSS Inc., Chicago, IL, USA). We calculated intrarater reliability for the placed landmarks and interrater reliability to compare reliability between the three sessions of placing landmarks. Data regarding intra- and interrater reliability were analyzed with intraclass correlation coefficients (ICC) with acceptable reliability criteria > 0.75 [12]. Using SPSS, the two-way random effects model was used; absolute agreement and single measures were used. The results of all the extractions and calculations of the groups were compared, and the mean values (of range) of each patient group are compared using one-way ANOVA and appropriate post hoc tests; Bonferroni correction was used with alpha = 0.05 (SPSS for Windows (Version 21, SPSS Inc., Chicago, IL, USA)).

## Results

### Repeatability and reproducibility

Locating of landmarks was done in triplicate by three raters. All raters achieved excellent intraclass correlation scores (0.994–1.000) and excellent interclass correlation scores (0.989–1.000).

### Overview curves

Figure 4c shows an example of an obtained curve. The curve starts at the occiput and skull outline is followed clockwise. After the first peak, resembling the occiput, the curve decreases, because the distance from the CM to the right side of the head is shorter than the distance from CM to the forehead or occiput. The second peak resembles the forehead; again the curve decreases to the left side of the head and increases to the occiput (Fig. 4c).

**Fig. 5:**
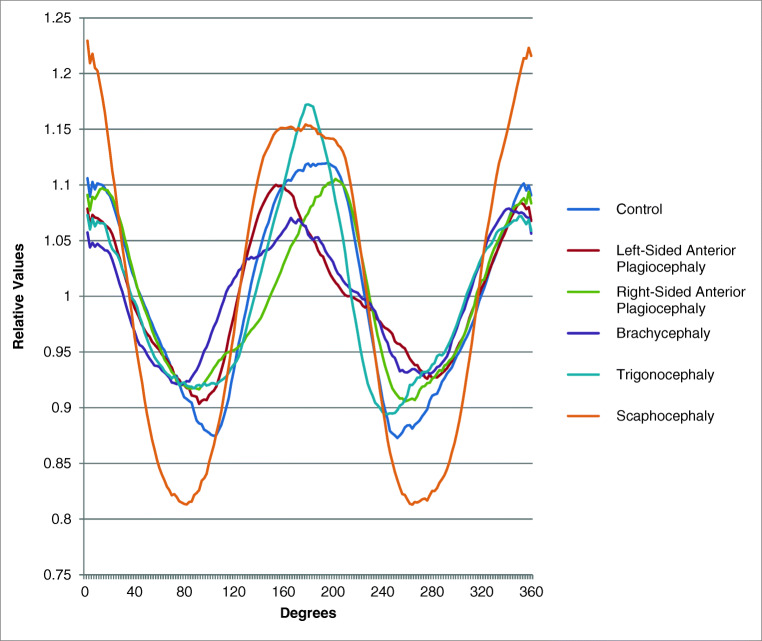
Overview mean graphs of all patient groups and controls

Figure 5 shows an overview of curves of the mean values of each subgroup. Each subgroup includes 5 patients; patient characteristics can be found in Table 2. For all patients, variables are extracted and calculated using the resulting curves. Only notable characteristics of the curves of each subgroup will be discussed.

**Table 2:** Patient Characteristics

Patient Group	Age (months) (mean (min. – max.))	Sex (male vs. female)
Control patients	49 (37–66)	5 vs. 0
Scaphocephaly	5.2 (2–9)	5 vs. 0
Trigonocephaly	4.6 (1–8)	3 vs. 2
Anterior plagiocephaly	7.9 (2–18)	1 vs. 9
Brachycephaly	3.8 (2–5)	1 vs. 4

### Control patients

The length of the skull is 30.5% longer than the width of the skull. Peaks of the forehead and occiput are of equal height. Mean width of forehead ratio is 78.61 (64.99–92.91). Mean asymmetry ratio is 1.05 (0.93–1.26), with the peak of the forehead in the middle of the curve (180° ± 12).

### Scaphocephaly

Both the forehead (1.16) and occiput are relatively long (1.24: the longest of all groups); the difference is − 0.08. The width is relatively small (mean = 0.81, the lowest of groups). The length of the skull is 48.5% longer than the width of the skull. The difference between the occiput and sides of the head is the highest (0.43). Mean width of forehead ratio is 84.59 (73.60–98.04). Mean asymmetry ratio is 1.02 (0.96–1.09), with the peak of the forehead in the middle of the curve (180° ± 12).

### Trigonocephaly

The forehead is relatively long (1.18 = highest mean), the occiput is slightly shorter (1.09), and the difference is 0.09. Width is normal (mean = 0.89), and the length of the skull is 27.2% longer than the width. Difference between the occiput and sides of the head is slightly less than normal (0.20). Mean width of forehead ratio is 47.95 (34.33–65.95), and mean asymmetry ratio is 1.39 (1.31–1.52), with the peak of the forehead in the middle of the curve (180° ± 12).

### Anterior plagiocephaly

Both the forehead (1.11 RS and 1.11 LS) and occiput (1.11 RS and 1.09 LS) are slightly short. Difference is 0.01 RS and 0.02 LS. Width is broader (mean 0.90 RS and 0.91 LS). The length of the skull is 23.0% RS and 20.6% LS longer than width in RS and LS. Difference between the occiput and sides of the head is slightly less than normal (0.20 RS and 0.18 LS). Mean width of forehead ratio is 83.44 (49.99–131.75) in RS and 96.42 (59.77–172.94) in LS. Mean asymmetry ratio is 1.82 (1.32–2.43) in RS and 0.55 (0.41–0.70) in LS, with the peak of the forehead deviated > 12° less than 180° (< 168°) RS and > 12° more than 180° (> 192°) LS.

### Brachycephaly

Both the forehead (1.08 = lowest mean) and occiput are short (1.09 = lowest mean); the difference is 0.00, meaning the peaks of the forehead and occiput are of equal height. The width is high (mean = 0.91). The length of the skull is 19.5% longer than width. Difference between the occiput and sides of the head is less than normal (0.18 = lowest mean). Mean width of forehead ratio is 123.02 (68.72–169.14). The asymmetry ratio is 1.11 (0.93–1.21), with the peak of the forehead in the middle of the curve (180° ± 12).

### Comparison of means

Mean difference between maximum and minimum values (i.e., range) in the curve for each degree in control patients was 0.12 (SD 0.04), in scaphocephaly 0.08 (SD 0.02), in trigonocephaly 0.08 (SD 0.03), in LS 0.07 (SD 0.02) and in RS 0.08 (SD 0.02) anterior plagiocephaly and in brachycephaly 0.07 (SD 0.03).

When comparing mean differences, one-way ANOVA showed significant difference between subgroups (*p* < 0.001). Following, Levene’s test showed assumption of homogeneity of variances between the groups was violated (*p* < 0.001). Therefore, Games-Howell test was performed as post hoc test and additionally Bonferroni correction. This showed the mean of control group was significantly higher than in all other patient groups (*p* < 0.001). Also, significant differences in means between the left-sided anterior plagiocephaly and right-sided anterior plagiocephaly, trigonocephaly, and scaphocephaly (all *p* < 0.001) were found.

## Discussion

In the present study, we introduced a new methodology for analyzing and diagnosing skull deformities using external landmarks and a two-dimensional skull shape outline. Until now, no accurate method of measurement for skull shape was available applicable to all types of craniosynostosis. Additionally, there was no valid comparative method for skull shape. Therefore, the purpose of this study was to develop a new method of measurement that makes comparative analysis possible in both craniosynostosis and control patients.

As stated before, currently widely used methods of measurement are CI and head circumference [3, 8, 9]. Other proposed methods are SSI, CVAI, and PCM [6, 10, 11]. However, these latter methods are only applicable for scaphocephaly, anterior plagiocephaly, or positional skull deformations and therefore not generalizable to most common craniosynostosis diagnoses.

When assessing the potential for proper classification of different types of craniosynostosis, a key issue arises. Both CI and CVAI are widely used in clinical settings, since they are fast, cheap, and easily applicable [13, 14]. However, measurements and calculations only using greatest width and greatest length (CI) or cranial diagonal diameters (CVAI) do not capture the actual shape of deformity, and additional quantification is necessary [6, 10, 15].

Another well-established morphometric parameter for skull growth is head circumference. The measurement is taken around the largest part of the head, above the eyebrows, above the ears, and the most posterior part of the head. It is another fast, easy, and cheap method but also discarding other features of dysmorphology [9].

ICV is another used method; however, volume gives no additional information about the skull shape and in craniosynostosis, compensatory growth will occur in a direction parallel to a fused suture and this explains why ICV is often within normal range in children with craniosynostosis [8, 16].

An outline-based approach, such as our proposed method, has the advantage that it can account for complex geometric variations in shape. External landmarks were chosen, in order to evolve our method to using 3D photogrammetry in the future. Using 3D photogrammetry, there is no need to expose the young patient to irradiation (CT scans) or general anesthesia (MRI) and its consequences [7, 17–20]. Accuracy of anthropometry is depending on the accurate identification and reliability of anatomical landmarks. The used exocanthion and porion are external landmarks, clearly identifiable without palpation, and are widely accepted [9]. Based on our consensus, we used LS and RS exocanthion and LS porion. However, in left-sided plagiocephaly, we used RS porion, because this side was unaffected by the skull deformity. Slices at 4 cm height gave the most proper outline of the skull without orbital disturbance and were used.

We calculated repeatability and reproducibility of our proposed methodology. This is a first step in validation, and further validation is essential in implementing a new approach for classification of congenital skull malformations. Based on the selected external landmarks, we found both excellent repeatability and reproducibility (intra- and interrater reliability) and this method can therefore be used to create valid two-dimensional slice images for skull shape outline.

We determined if the peak of the forehead is around 180° ± 12°. This value of 12° is supported by our results and corresponds to the used value of asymmetry of > 3.5% in the CVAI (3.5% of 360° corresponds to a value of 12.6), which shows significantly asymmetrical values of the head in plagiocephaly patients [10]. A notable finding in this study is the asymmetry ratio of trigonocephaly patients (Fig. 5). In these patients, the peak of the forehead is located at 180° ± 12°; however, the relatively high asymmetry ratio shows values comparable with right-sided anterior plagiocephaly. In future research, we will focus on the cause of this finding, e.g. coexisting positional plagiocephaly or incidental finding.

Range between maximum and minimum values of curves is (statistically significant) larger in the control group than in other patient groups. This can be explained by a (wide) normal variation in skull shapes in normal children, whereas craniosynostosis patients have growth restriction in one direction and accelerated compensatory growth in the perpendicular direction.

We have presented a new approach for diagnosing different types of craniosynostosis. We can conclude that every type of craniosynostosis has a specific and recognizable skull deformity, and therefore we can identify a trend towards a specific and characteristic pattern of the curve for the different types. Based on the curve and values contributing to the curve, our novel method is a promising tool in diagnosing craniosynostosis. This method could be a useful tool in the field of research of craniosynostosis and in the clinical setting. By using external landmarks, future research using 3D photogrammetry to create a 2D skull outline is a promising non-invasive alternative, which can be used for monitoring growth and surgical results and potentially quantifying severity of craniosynostosis. However, further research is necessary with a larger group of patients in order to further analyze and validate our method and to be generalizable for a larger group of patients.

## Abbreviations

3D: Three-dimensional
CI: Cranial index
CM: Center of mass
CT: Computed tomography
CVAI: Cranial vault asymmetry index
Ex: Exocanthion
F: Forehead peak
ICC: Intraclass correlation coefficients
ICV: Intracranial volume
L: Minimum value of the left side of the head
LS: Left-sided
MRI: Magnetic resonance imaging
O: Occiput peak
PCM: Plagiocephalometry
Po: Porion
R: Minimum value of the right side of the head
RS: Right-sided
SP: Starting point
SSI: Scaphocephaly severity index
XF: *X*-value of maximum forehead value
XFL: *X*-value for the maximum forehead minus 0.1 on the left side
XFR: *X*-value for the maximum forehead minus 0.1 on the right side
XL: *X*-value of the minimum value of the width on the left side
XR: X-value of the minimum value of the width on the right side
